# Supplementary material for: Clostridioides difficile Toxin B PCR Cycle Threshold as a Predictor of Toxin Testing in Stool Specimens from Hospitalized Adults
Source: Antibiotics (Basel). 2022 Apr 26;11(5):576. doi: 10.3390/antibiotics11050576 (PMC9137712; doi:10.3390/antibiotics11050576)
Supplement: Supplementary file 1 [file antibiotics-11-00576-s001.zip › antibiotics-1686116-supplementary.pdf]

Date	Age	Gender	Admit category	GDH result	Toxin EIA result	CCNA result	Cdiff PCR tcdB Ct	Cdiff PCR bin tox Ct	Cdiff PCR tcdc del Ct
2016/12/10	63	M	cancer	Positive	Negative	Negative	27.9	27.2	0
2016/12/10	60	F	hem malignancy	Negative	Negative	Negative	36.9	0	0
2016/12/12	58	F	SOT	Positive	Negative	Negative	25.8	0	0
2016/12/14	35	M	BMT	Positive	Positive	Positive	26.1	25.8	26.5
2016/12/14	76	M	hem malignancy	Negative	Negative	Negative	36.9	0	0
2016/12/16	54	M	surgery	Positive	Positive	Positive	25.2	24.1	0
2016/12/19	47	F	surgery CV	Positive	Negative	Negative	35.5	0	0
2016/12/21	58	M	SOT	Positive	Negative	Positive	27.9	0	0
2016/12/23	69	F	NS	Positive	Negative	Positive	31.0	0	0
2016/12/27	55	M	CV	Positive	Negative	Positive	32.8	0	0
2017/1/7	58	M	BMT	Positive	Negative	Negative	30.2	0	0
2017/1/12	43	M	BMT	Positive	Positive	Positive	23.1	0	0
2017/1/14	71	M	surgery GI	Positive	Negative	Positive	24.7	0	0
2017/1/18	33	F	infection	Positive	Negative	Negative	34.6	0	0
2017/1/25	50	F	cancer	Negative	Negative	Negative	29.0	0	0
2017/2/3	71	F	cancer	Positive	Positive	Positive	25.6	0	0
2017/2/3	61	F	surgery GI	Positive	Positive	Positive	23.4	25.2	25.8
2017/2/5	56	F	cancer	Positive	Positive	Positive	22.5	21.8	0
2017/2/6	75	M	GI bleed	Positive	Negative	Negative	29.7	0	0
2017/2/7	40	M	SOT	Positive	Negative	Positive	31.9	31.1	32.1
2017/2/18	66	M	cancer	Positive	Positive	Positive	34.6	34	35
2017/2/23	56	F	hem malignancy	Negative	Negative	Negative	32.6	0	0
2017/2/23	72	F	infection	Positive	Negative	Positive	32.6	0	0
2017/3/7	60	M	surgery CV	Positive	Positive	Positive	23.4	0	0
2017/3/8	72	M	NS	Positive	Negative	Positive	22.5	0	0.0
2017/3/15	75	F	cancer	Positive	Positive	Positive	25.1	24.4	0
2017/3/23	71	F	cancer	Positive	weak pos	Positive	23.9	0	0
2017/3/24	77	F	GI	Positive	Positive	Positive	21.3	20.5	21.4
2017/4/7	55	M	renal failure	Positive	Negative	Positive	32.2	31.3	32.3
2017/4/18	70	F	cancer	Positive	Negative	Negative	26.7	0	0
2017/4/19	81	F	rheum	Positive	Negative	Positive	27.2	0	0
2017/4/26	47	M	cancer	Positive	Negative	Positive	26.4	0	0
2017/5/1	55	F	cancer	Positive	Positive	Positive	23.4	0	0
2017/5/2	66	M	SOT	Positive	Positive	Positive	22.4	0	0
2017/5/5	56	M	SOT	Positive	Negative	Positive	26.3	0	0
2017/5/12	44	F	pulm	Negative	Negative	Negative	32.9	0	0
2017/5/19	52	M	CV	Positive	Negative	Positive	29.1	0	0
2017/5/23	39	M	BMT	Positive	Negative	Positive	24.6	0	0
2017/5/23	56	F	cancer	Positive	Positive	Positive	23.2	0	0
2017/5/26	71	M	hem malignancy	Positive	Negative	Positive	24.3	0	0
2017/5/28	71	M	cancer	Positive	Positive	Positive	28.1	27.1	27.9
2017/5/30	84	F	neoplasm	Positive	Negative	Negative	21.4	0	0
2017/6/2	41	M	BMT	Positive	Negative	Positive	21.5	0	0
2017/6/2	32	M	BMT	Positive	Positive	Positive	24.5	0	0
2017/6/3	39	F	hem malignancy	Positive	Negative	Positive	29.1	30.7	0
2017/6/9	65	F	surgery GI	Positive	weak pos	Positive	26.2	0	0
2017/6/12	54	M	SOT	Positive	Negative	Negative	27.2	0	0
2017/6/13	63	M	NS	Positive	Positive	Positive	26.0	0	0
2017/6/13	84	F	renal failure	Positive	Positive	Positive	21.3	25.6	0

2017/6/15	55	F	cancer	Negative	Negative	Positive	30.5	0	0
2017/6/15	53	F	pulm	Positive	Negative	Negative	32.8	0	0
2017/6/16	82	F	NS	Positive	Positive	Positive	20.8	0	0
2017/6/21	70	F	hem malignancy	Positive	Negative	Positive	28.2	0	0
2017/6/30	62	M	GI bleed	Positive	Positive	Positive	25.7	0	0
2017/7/3	58	F	NS	Negative	Negative	Positive	36.8	0	0
2017/7/3	60	M	surgery GI	Positive	Negative	Positive	28.4	0	0
2017/7/7	75	M	cancer	Positive	Positive	Positive	22.5	22.1	22.1
2017/7/9	67	M	liver failiure	Positive	Positive	Positive	22.8	22.4	22.6
2017/7/16	68	M	NS	Positive	Positive	Positive	31.2	0	0
2017/7/21	73	M	IBD	Negative	Negative	Negative	34.8	0	0
2017/7/21	57	M	SOT	Positive	Negative	Positive	29.8	0	0
2017/7/24	29	F	BMT	Positive	Negative	Negative	29.3	0	0
2017/7/24	72	F	SOT	Positive	Negative	Negative	31.5	0	0
2017/7/25	51	F	cancer	Positive	Negative	Negative	36.6	0	0
2017/7/26	50	M	cancer	Positive	Negative	Negative	27.1	0	0
2017/8/1	75	F	cancer	Positive	Negative	Positive	25.1	0	0
2017/8/1	85	M	surgery GU	Positive	Negative	Negative	29.7	0	0
2017/8/4	80	F	CV	Positive	Negative	Negative	30.3	0	0
2017/8/5	63	M	cancer	Positive	Negative	Positive	28.5	0	0
2017/8/5	54	F	NS	Positive	weak pos	Positive	36.3	0	0
2017/8/14	25	F	BMT	Negative	Negative	Negative	25.3	0	0
2017/8/14	77	M	cancer	Positive	Negative	Positive	27.1	0	0
2017/8/22	66	M	cancer	Positive	Negative	Positive	34.9	0	0
2017/8/26	51	M	CV	Positive	Positive	Positive	28.5	0	0
2017/8/31	84	M	NS	Positive	Negative	Positive	26.0	0	0
2017/9/2	68	F	BMT	Positive	Negative	Positive	31.7	0	0
2017/9/2	63	F	cancer	Negative	Negative	Negative	34.3	0	0
2017/9/4	64	M	NS	Positive	Positive	Positive	21.1	0	0
2017/9/5	56	F	BMT	Positive	Negative	Negative	24.6	0	0
2017/9/6	38	M	IBD	Positive	Negative	Negative	27.1	0	0
2017/9/20	48	F	cancer	Positive	Negative	Positive	26.9	0	0
2017/9/25	62	F	surgery GI	Positive	Negative	Positive	26.3	0	0
2017/9/29	50	F	cancer	Positive	Negative	Negative	35.3	0	0
2017/9/29	33	F	CV	Positive	Negative	Positive	31.1	0	0
2017/10/2	65	F	liver failiure	Positive	Negative	Positive	27.5	0	0
2017/10/3	76	F	CV	Positive	Positive	Positive	23.8	0	0
2017/10/9	75	F	cancer	Negative	Negative	Negative	35.4	0	0
2017/10/23	54	M	cancer	Positive	Negative	Positive	36.2	36.1	36.4
2017/10/29	25	M	GI	Positive	Negative	Negative	26.1	0	0
2017/11/3	72	M	IBD	Positive	Negative	Negative	27.5	0	0
2017/11/12	74	F	surgery GI	Positive	Negative	Negative	23.6	0	0
2017/11/14	47	F	hem malignancy	Positive	Negative	Negative	36.8	0	0
2017/11/17	59	F	cancer	Positive	Negative	Positive	32.2	0	0
2017/11/20	54	F	cancer	Positive	Positive	Positive	25.2	0	0
2017/11/21	24	F	IBD	Negative	Negative	Negative	36.2	0	0
2017/11/27	45	F	cancer	Positive	Negative	Negative	29.5	0	0
2017/11/29	70	F	hem malignancy	Positive	Negative	Negative	34.4	0	0
2017/12/3	58	M	hem malignancy	Positive	Negative	Positive	19.3	0	0
2017/12/18	31	M	CV	Positive	Negative	Positive	32.9	0	0
2017/12/23	56	M	SOT	Positive	Negative	Positive	27.1	0	0

2017/12/25	56	F	cancer	Positive	Positive	Positive	22.6	0	0
2017/12/30	75	M	cancer	Negative	Negative	Negative	32.3	0	0
2018/1/8	77	F	SOT	Positive	Negative	Positive	32.9	0	0
2018/1/10	65	M	GI bleed	Positive	Negative	Positive	30.1	29.7	29.7
2018/1/14	53	M	liver failiure	Positive	Negative	Positive	32.5	32.1	32.6
2018/1/17	39	M	liver failiure	Positive	Positive	Positive	27.2	26.9	26.6
2018/1/22	69	F	cancer	Positive	Negative	Negative	35.8	0	0
2018/1/23	66	F	BMT	Positive	Negative	Negative	32.4	0	0
2018/1/26	81	M	infection	Positive	Negative	Positive	30.9	0	0
2018/1/29	56	F	CV	Positive	Positive	Positive	28.0	0	0
2018/2/8	54	F	surgery CV	Positive	Negative	Positive	25.1	0	0
2018/2/12	85	M	surgery GI	Positive	Positive	Positive	23.8	24.1	24.6
2018/2/13	63	M	liver failiure	Negative	Negative	Negative	31.0	0	0
2018/2/15	47	M	GI	Positive	Negative	Negative	34.5	0	0
2018/2/16	85	F	cancer	Positive	Positive	Positive	24.6	24.3	24.4
2018/2/20	54	M	surgery GI	Positive	Positive	Positive	26.0	0	0
2018/2/24	75	M	surgery CV	Positive	Negative	Positive	30.6	29.9	0
2018/3/3	55	F	BMT	Positive	Negative	Negative	29.5	0	0
2018/3/28	23	F	BMT	Positive	Negative	Positive	28.4	27.8	28.2
2018/3/29	66	F	CV	Positive	Negative	Positive	26.6	0	0
2018/4/1	79	M	CV	Positive	Positive	Positive	20.8	20.1	20.5
2018/4/3	66	M	CV	Positive	Positive	Positive	24.5	0	0
2018/4/5	68	F	cancer	Positive	Positive	Positive	29.8	0	0
2018/4/7	77	F	GI	Positive	Negative	Positive	33.4	0	0
2018/4/10	71	F	SOT	Positive	Positive	Positive	23.8	23.7	23.3
2018/4/14	19	M	BMT	Positive	Negative	Positive	25.6	0	0
2018/4/20	50	F	renal failure	Positive	Negative	Negative	29.1	0	0
2018/4/22	75	M	cancer	Positive	Positive	Positive	22.0	0	0
2018/4/23	83	M	CV	Positive	Positive	Positive	30.1	0	0
2018/4/27	51	M	BMT	Positive	Positive	Positive	23.8	0	0
2018/4/27	79	F	cancer	Positive	Positive	Positive	27.4	0	0
2018/5/1	51	F	NS	Positive	Positive	Positive	26.0	0	0
2018/5/2	68	F	BMT	Positive	Negative	Positive	33.6	0	0
2018/5/4	59	F	surgery GI	Positive	Negative	Negative	22.5	22	22.3
2018/5/6	79	M	cancer	Positive	Positive	Positive	26.3	0	0
2018/5/6	33	M	NS	Positive	Negative	Negative	34.6	0	0
2018/5/12	64	M	cancer	Positive	Positive	Positive	21.3	0	0
2018/5/14	44	M	BMT	Positive	Negative	Positive	30.8	0	0
2018/5/16	65	F	cancer	Positive	Negative	Positive	35.1	0	0
2018/5/18	66	F	cancer	Positive	Negative	Negative	35.4	0	0
2018/5/23	87	M	cancer	Positive	Positive	Positive	27.8	27.2	27.3
2018/5/25	65	F	GI bleed	Negative	Negative	Negative	32.5	0	0
2018/5/29	63	F	SOT	Positive	Negative	Positive	23.5	0	0
2018/6/5	76	F	surgery ortho	Positive	Positive	Positive	23.2	0	0
2018/6/9	77	M	CV	Positive	Negative	Negative	35.4	34.8	36
2018/6/10	61	M	surgery GU	Positive	Negative	Negative	34.0	0	0
2018/6/14	22	M	IBD	Positive	Positive	Positive	23.2	0	0
2018/6/14	72	M	SOT	Positive	Positive	Positive	36.1	35.2	35.4
2018/6/15	57	F	cancer	Positive	Positive	Negative	35.0	0	0
2018/6/17	32	F	NS	Positive	Positive	Positive	20.3	19.7	19.9
2018/6/22	56	M	SOT	Positive	Negative	Positive	23.5	0	0

2018/6/25	37	M	BMT	Positive	Negative	Positive	32.0	0	0
2018/6/30	66	F	liver failiure	Positive	Negative	Positive	29.5	0	0
2018/7/2	54	F	surgery ortho	Positive	Negative	Positive	25.2	0	0
2018/7/10	31	M	SOT	Negative	Negative	Negative	34.1	0	0
2018/7/13	81	F	cancer	Positive	Negative	Negative	25.1	0	0
2018/7/15	65	F	SOT	Positive	Negative	Positive	30.6	0	0
2018/7/17	64	F	IBD	Positive	Negative	Negative	24.7	24.3	24.3
2018/7/19	65	F	neoplasm	Positive	Negative	Positive	24.7	0	0
2018/7/24	55	F	CV	Positive	Positive	Positive	23.7	23.2	23.3
2018/7/24	67	M	surgery GI	Positive	Negative	Negative	28.5	0	0
2018/8/1	73	M	surgery ortho	Positive	Positive	Positive	24.8	24.2	24.5
2018/8/5	67	F	surgery CV	Positive	Negative	Positive	31.4	31.3	31.2
2018/8/6	55	M	liver failiure	Positive	Negative	Positive	34.4	33.4	0
2018/8/10	33	F	surgery GI	Positive	Negative	Negative	25.6	0	0
2018/8/12	48	F	liver failiure	Positive	Negative	Negative	28.3	0	0
2018/8/12	35	F	NS	Negative	Negative	Positive	32.0	0	0
2018/8/16	69	F	cancer	Negative	Negative	Positive	34.4	0	0
2018/8/24	73	M	surgery ortho	Positive	Positive	Positive	31.2	30.2	30.3
2018/9/6	70	F	surgery	Positive	Positive	Positive	21.8	21.3	21.6
2018/9/12	66	M	cancer	Positive	Negative	Positive	23.7	0	0
2018/9/14	93	M	surgery GI	Positive	Negative	Positive	25.1	24.6	25
2018/9/17	67	M	BMT	Positive	Negative	Negative	30.5	0	0
2018/9/19	67	M	cancer	Positive	Negative	Negative	28.7	0	0
2018/9/30	60	M	hem malignancy	Positive	Negative	Positive	20.8	0	0
2018/10/1	57	M	cancer	Positive	Negative	Negative	31.9	31.6	0
2018/10/7	77	F	CV	Positive	Negative	Positive	30.6	0	0
2018/10/14	65	M	liver failiure	Positive	Positive	Positive	20.5	20.2	20.6
2018/10/14	53	F	SOT	Positive	Negative	Positive	30.1	0	0
2018/10/16	47	F	SOT	Positive	Negative	Negative	28.5	0	0
2018/10/17	50	F	CV	Positive	Negative	Positive	20.1	0	0
2018/10/20	83	M	cancer	Positive	Positive	Positive	29.2	0	0
2018/10/26	64	M	SOT	Negative	Negative	Negative	32.9	0	0
2018/11/3	57	F	hem malignancy	Positive	Negative	Positive	33.5	33.1	33.1
2018/11/14	66	M	cancer	Positive	Positive	Positive	25.7	0	0
2018/11/15	87	M	renal failure	Positive	Positive	Positive	32.4	26.9	0
2018/11/15	60	M	SOT	Positive	Negative	Positive	27.7	32.1	32.2
2018/11/17	47	M	BMT	Positive	Positive	Positive	20.8	0	0
2018/11/19	52	F	cancer	Positive	Positive	Positive	23.8	0	0
2018/11/19	65	F	surgery ortho	Positive	Positive	Positive	27.4	27	26.9
2018/11/22	43	M	BMT	Positive	Negative	Negative	34.7	33.5	33.8
2018/11/24	24	M	IBD	Positive	Positive	Positive	21.8	0	0
2018/11/24	70	F	NS	Positive	Negative	Negative	35.4	0	0
2018/11/29	76	F	neoplasm	Positive	Negative	Positive	26.6	0	0
2018/12/8	50	F	neoplasm	Positive	Positive	Positive	25.3	0	0
2018/12/8	66	M	NS	Positive	Positive	Positive	24.8	0	0
2018/12/8	62	F	surgery GU	Positive	Negative	Negative	24.8	24.4	0
2018/12/9	64	F	BMT	Positive	Positive	Positive	25.6	0	0
2018/12/9	62	M	liver failiure	Negative	Negative	Positive	34.1	0	0
2018/12/10	38	F	cancer	Positive	Negative	Negative	32.5	0	0
2018/12/12	57	F	cancer	Positive	Negative	Negative	30.4	0	0
2018/12/19	64	F	cancer	Positive	Positive	Positive	28.6	27.6	0

2018/12/22	28	M	BMT	Positive	Negative	Positive	20.7	0	0
2018/12/24	70	M	surgery CV	Positive	Positive	Positive	22.7	0	0
2019/1/2	62	M	SOT	Negative	Negative	Positive	34.4	0	0
2019/1/6	29	F	SOT	Positive	Negative	Positive	29.2	0	0
2019/1/8	63	F	surgery CV	Positive	Negative	Positive	28.4	0	0
2019/1/11	70	M	surgery CV	Positive	Positive	Positive	31.2	31.7	31.7
2019/1/14	75	M	cancer	Negative	Negative	Positive	32.9	0	0
2019/1/19	61	M	cancer	Positive	Negative	Positive	24.3	0	0
2019/1/22	69	M	pulm	Positive	Negative	Positive	29.7	29.6	0
2019/1/25	40	M	liver failiure	Positive	Positive	Positive	28.1	27.6	27.4
2019/1/28	67	F	surgery CV	Positive	Negative	Negative	32.4	0	0
2019/1/29	58	M	surgery CV	Positive	Positive	Positive	26.5	0	0
2019/2/4	29	F	SOT	Positive	Negative	Negative	23.8	0	0
2019/2/8	55	F	surgery GU	Positive	Negative	Positive	25.8	0	0
2019/2/10	65	M	hem malignancy	Positive	Positive	Positive	25.0	0	0
2019/2/11	67	M	SOT	Positive	Negative	Positive	22.7	0	0
2019/2/13	62	M	surgery GI	Positive	Negative	Positive	28.9	28.1	28.2
2019/2/21	50	M	surgery CV	Positive	Negative	Positive	28.7	0	0
2019/2/27	52	F	CV	Positive	Positive	Positive	24.9	24.2	24.1
2019/3/2	60	M	SOT	Positive	Negative	Positive	23.4	0	0
2019/3/8	82	M	surgery GI	Positive	Negative	Positive	33.1	0	0
2019/3/16	19	F	surgery GI	Positive	Negative	Positive	31.2	0	0
2019/3/17	79	M	surgery GI	Positive	Negative	Positive	30.0	0	0
2019/3/17	64	F	surgery ortho	Positive	Negative	Positive	20.5	0	0
2019/3/23	63	F	cancer	Positive	Negative	Positive	30.2	0	0
2019/3/26	44	F	cancer	Negative	Negative	Negative	35.0	34.7	0
2019/3/28	71	F	SOT	Positive	Negative	Negative	34.4	0	0
2019/4/4	73	M	IBD	Negative	Negative	Negative	35.1	0	0
2019/4/6	21	F	IBD	Positive	Positive	Positive	21.1	0	0
2019/4/6	24	M	SOT	Positive	Negative	Positive	26.0	0	0
2019/4/8	75	M	cancer	Positive	Positive	Positive	24.1	0	0
2019/4/8	57	M	SOT	Positive	Negative	Negative	34.7	0	0
2019/4/9	44	F	cancer	Positive	Negative	Negative	34.3	34.1	0
2019/4/19	74	M	surgery GI	Positive	Negative	Negative	32.3	0	0
2019/4/21	43	M	surgery CV	Positive	Negative	Positive	23.5	0	0
2019/4/27	29	M	surgery	Positive	Negative	Positive	23.2	24.7	0
2019/4/29	76	M	surgery GI	Positive	Negative	Positive	27.5	0	0
2019/5/2	74	M	GI	Positive	Negative	Positive	20.8	0	0
2019/5/6	29	F	SOT	Negative	Negative	Negative	33.4	0	0
2019/5/23	66	M	surgery CV	Positive	Negative	Positive	26.8	0	0
2019/5/25	43	M	SOT	Positive	Positive	Positive	27.1	0	0
2019/6/1	26	F	IBD	Positive	Negative	Negative	28.0	0	0
2019/6/6	74	F	hem malignancy	Positive	Positive	Positive	22.1	0	0
2019/6/8	38	F	NS	Positive	Positive	Positive	25.2	0	0
2019/6/23	73	F	cancer	Positive	Negative	Positive	28.1	27.5	0
2019/7/1	76	M	NS	Positive	Positive	Positive	23.8	0	0
2019/7/5	79	M	cancer	Positive	Negative	Positive	24.0	0	0
2019/7/16	41	M	liver failiure	Positive	Negative	Positive	32.4	0	0
2019/7/24	73	F	surgery GI	Positive	Negative	Positive	28.7	0	0
2019/8/5	25	F	SOT	Positive	Positive	Positive	27.6	0	0
2019/8/15	46	F	cancer	Positive	Negative	Negative	22.4	0	0

2019/8/20	42	F	cancer	Positive	Negative	Positive	28.4	32.3	31.7
2019/8/28	58	F	cancer	Positive	Positive	Positive	23.1	0	0
2019/9/1	81	M	cancer	Positive	Negative	Negative	29.7	0	0
2019/9/2	51	M	NS	Positive	Positive	Positive	23.1	0	0
2019/9/3	72	M	surgery GI	Positive	Positive	Positive	27.1	26.7	0
2019/9/5	62	F	surgery CV	Positive	Negative	Positive	28.4	0	0
2019/9/6	81	M	hem malignancy	Positive	Positive	Positive	24.9	24.7	24.2
2019/9/7	75	F	surgery CV	Positive	Positive	Positive	22.1	0	0
2019/9/10	50	F	CV	Positive	Negative	Positive	25.6	0	22.7
2019/9/10	36	M	SOT	Positive	Positive	Positive	23.3	0	0
2019/9/10	60	F	SOT	Negative	Negative	Negative	31.2	0	0
2019/9/13	71	M	cancer	Positive	Negative	Positive	27.3	0	0
2019/9/21	36	M	SOT	Positive	Negative	Negative	36.8	0	37.1
2019/9/22	50	F	cancer	Positive	Positive	Positive	35.3	0	0
2019/9/24	79	M	cancer	Positive	Positive	Positive	29.2	0	0
2019/9/26	72	M	surgery GI	Positive	Positive	Positive	24.3	23.8	0
2019/9/27	65	F	cancer	Positive	Negative	Positive	30.0	0	0
2019/10/1	30	F	SOT	Positive	Negative	Positive	27.1	0	0
2019/10/4	55	F	SOT	Positive	Negative	Negative	35.7	0	0
2019/10/13	38	M	liver failiure	Negative	Negative	Positive	30.4	0	0
2019/10/15	59	F	cancer	Positive	Negative	Positive	29.8	0	0
2019/10/20	70	M	cancer	Positive	Positive	Positive	27.7	0	0
2019/10/23	32	F	SOT	Negative	Negative	Negative	32.9	0	0
2019/10/29	60	F	NS	Positive	Positive	Positive	28.6	0	0
2019/10/30	65	F	surgery GI	Positive	Negative	Negative	33.7	0	0
2019/11/1	59	M	cancer	Positive	Positive	Positive	23.2	0	0
2019/11/5	47	M	SOT	Positive	Positive	Negative	27.9	0	0
2019/11/7	22	F	rheum	Positive	Positive	Positive	24.5	0	0
2019/11/10	50	M	SOT	Positive	Positive	Negative	26.1	0	0
2019/11/12	71	M	SOT	Positive	Negative	Negative	29.8	0	0
2019/11/13	75	F	surgery CV	Positive	Negative	Negative	30.3	32.5	34.8
2019/11/14	64	M	surgery	Positive	Positive	Positive	25.7	0	0
2019/12/6	67	M	surgery GI	Positive	Positive	Positive	24.8	0	0
2019/12/8	58	M	NS	Positive	Positive	Positive	22.5	22.3	22.1
2019/12/10	84	M	cancer	Positive	Negative	Positive	35.2	0	0
2019/12/15	72	M	cancer	Positive	Positive	Positive	20.2	0	0
2019/12/27	64	F	SOT	Positive	Positive	Positive	24.5	0	0
2019/12/31	39	F	SOT	Positive	Positive	Positive	23.9	23.7	23.1
2020/1/10	63	M	SOT	Positive	Negative	Positive	32.2	0	0
2020/1/20	40	M	surgery CV	Positive	Negative	Positive	20.3	0	0
2020/2/5	55	M	surgery CV	Positive	Negative	Negative	29.7	26.3	0
2020/2/6	49	M	SOT	Positive	Positive	Positive	25.7	0	0
2020/2/7	64	F	SOT	Positive	Negative	Negative	35.3	0	0
2020/2/8	23	F	surgery GI	Positive	Negative	Negative	29.1	31.6	0
2020/2/9	80	F	cancer	Positive	Negative	Positive	25.4	0	0
2020/2/10	38	M	cancer	Positive	Negative	Negative	26.2	0	0
2020/2/10	78	M	cancer	Negative	Negative	Negative	26.7	0	0
2020/2/18	65	F	surgery CV	Positive	Negative	Positive	27.2	0	0
2020/2/20	59	F	SOT	Negative	Negative	Negative	32.6	0	0
2020/2/20	70	F	surgery	Negative	Negative	Negative	35.1	0	0
2020/2/25	34	M	SOT	Positive	Negative	Negative	28.1	0	0

2020/2/27	50	F	IBD	Positive	Positive	Positive	23.4	23.1	23
2020/2/27	49	M	liver failiure	Negative	Negative	Negative	36.1	0	0
2020/3/1	49	M	SOT	Positive	Positive	Positive	22.4	0	0
2020/3/1	42	M	SOT	Positive	Negative	Positive	34.1	33.6	0
2020/3/7	75	M	cancer	Positive	Negative	Negative	23.3	22.7	22.7
2020/3/12	49	F	cancer	Positive	Positive	Positive	21.6	0	0
2020/3/16	60	M	SOT	Positive	Negative	Negative	22.8	22.7	0
2020/3/17	24	F	surgery GI	Positive	Negative	Negative	29.2	0	0
2020/3/22	43	M	SOT	Positive	Negative	Positive	25.8	0	0
2020/3/27	59	M	surgery GI	Positive	Negative	Positive	30.3	0	0
2020/4/2	66	M	liver failiure	Positive	Positive	Positive	21.1	0	0
2020/4/3	63	M	surgery CV	Positive	Positive	Positive	29.3	0	0
2020/4/8	68	M	infection	Positive	Positive	Positive	21.6	21.2	21.1
2020/4/20	71	M	surgery	Positive	Positive	Positive	24.5	0	0
2020/4/22	59	M	surgery GI	Positive	Negative	Positive	29.1	0	0
2020/4/26	83	F	surgery	Positive	Negative	Negative	33.6	0	0
2020/4/27	79	F	SOT	Positive	Negative	Positive	33.1	0	0
2020/5/1	54	M	IBD	Positive	Negative	Negative	24.3	24.1	23.4
2020/5/6	43	M	SOT	Negative	Negative	Negative	36.9	0	0
2020/5/9	64	F	cancer	Positive	Positive	Positive	25.0	0	0
2020/5/13	54	M	SOT	Positive	Positive	Positive	22.0	21.3	21.4
2020/5/18	80	F	hem malignancy	Positive	Positive	Positive	23.6	0	0
2020/5/18	64	F	SOT	Positive	Positive	Positive	21.6	23.3	22.8
2020/5/19	74	M	NS	Positive	Positive	Positive	23.8	0	0
2020/5/23	68	F	surgery CV	Positive	Negative	Negative	33.1	0	0
2020/6/2	34	M	cancer	Negative	Negative	Negative	32.8	0	0
2020/6/5	77	F	surgery CV	Positive	Negative	Negative	29.3	0	0
2020/6/8	67	F	liver failiure	Positive	Negative	Negative	34.6	0	0
2020/6/11	62	F	cancer	Positive	Positive	Positive	23.7	0	0
2020/6/13	71	F	CV	Positive	Negative	Positive	20.3	0	0
2020/6/15	61	F	cancer	Positive	Negative	Positive	32.2	32.5	0
2020/6/22	59	M	SOT	Positive	Positive	Positive	21.8	21.1	0
2020/6/25	65	F	cancer	Positive	Negative	Positive	34.2	0	0
2020/6/26	37	M	SOT	Positive	Positive	Positive	24.3	0	0
2020/6/29	62	F	NS	Negative	Negative	Negative	31.5	0	0
2020/7/1	70	F	cancer	Positive	Positive	Positive	25.6	0	0
2020/7/8	71	M	surgery	Negative	Negative	Negative	32.2	32.2	0
2020/7/17	51	M	cancer	Negative	Negative	Positive	31.0	0	0
2020/7/18	72	M	CV	Positive	Positive	Positive	24.7	0	0
2020/7/18	45	M	surgery CV	Positive	Positive	Negative	32.1	0	0
2020/7/20	67	M	SOT	Positive	Negative	Positive	21.8	0	0
2020/7/22	35	M	SOT	Positive	Negative	Positive	24.6	0	0
2020/7/22	33	F	SOT	Negative	Negative	Negative	33.9	0	0
2020/7/26	48	M	cancer	Positive	Negative	Positive	24.9	0	0
2020/7/26	69	F	SOT	Negative	Negative	Negative	34.9	0	0
2020/7/30	56	F	surgery CV	Positive	Negative	Negative	25.1	0	0
2020/8/7	65	F	cancer	Positive	Negative	Positive	27.2	0	0
2020/8/7	76	M	IBD	Positive	Negative	Positive	26.3	0	0
2020/8/10	48	M	liver failiure	Negative	Positive	Positive	25.3	24.8	24.6
2020/8/26	76	M	cancer	Positive	Positive	Positive	25.5	0	0
2020/8/26	44	M	SOT	Positive	Positive	Positive	25.9	0	0

2020/8/26	48	M	SOT	Negative	Negative	Negative	27.5	27.5	26.7
2020/8/28	72	M	surgery ortho	Positive	Positive	Positive	23.6	0	0
2020/8/29	57	M	cancer	Positive	Negative	Positive	33.3	0	0
2020/9/3	33	F	SOT	Positive	Negative	Negative	30.1	0	0
2020/9/8	26	M	IBD	Positive	Negative	Negative	24.2	23.8	24.5
2020/9/9	69	F	cancer	Positive	Negative	Positive	30.7	0	0
2020/9/11	23	F	surgery ortho	Positive	Positive	Positive	18.5	0	0
2020/9/26	67	F	surgery CV	Positive	Negative	Positive	19.3	0	0
2020/9/28	52	M	neoplasm	Positive	Negative	Positive	24.4	0	0
2020/10/3	66	M	surgery CV	Positive	Positive	Positive	24.1	0	0
2020/10/7	63	F	surgery GI	Positive	Negative	Positive	23.2	0	0
2020/10/8	56	F	IBD	Negative	Negative	Positive	26.0	0	0
2020/10/12	59	M	surgery GI	Positive	Negative	Positive	25.8	0	0
